# SHP2 as a Potential Therapeutic Target in Diffuse-Type Gastric Carcinoma Addicted to Receptor Tyrosine Kinase Signaling

**DOI:** 10.3390/cancers13174309

**Published:** 2021-08-26

**Authors:** Yuko Nagamura, Makoto Miyazaki, Yoshiko Nagano, Arata Tomiyama, Rieko Ohki, Kazuyoshi Yanagihara, Ryuichi Sakai, Hideki Yamaguchi

**Affiliations:** 1 Department of Cancer Cell Research, Sasaki Institute, Sasaki Foundation, Tokyo 101-0062, Japan; nagamura@po.kyoundo.jp (Y.N.); m-miyazaki@po.kyoundo.jp (M.M.); y-nagano@po.kyoundo.jp (Y.N.); 2 Department of Neurosurgery, National Defense Medical College, Saitama 359-8513, Japan; atomiyam@outlook.jp; 3 Laboratory of Fundamental Oncology, National Cancer Center Research Institute, Tokyo 104-0045, Japan; rohki@ncc.go.jp; 4 Division of Biomarker Discovery, Exploratory Oncology Research and Clinical Trial Center, National Cancer Center, Chiba 277-8577, Japan; kyanagih@east.ncc.go.jp; 5 Department of Biochemistry, Kitasato University School of Medicine, Sagamihara 252-0374, Japan; rsakai@kitasato-u.ac.jp

**Keywords:** diffuse-type gastric carcinoma, peritoneal dissemination, SHP2, receptor tyrosine kinase, Met

## Abstract

**Simple Summary:**

Diffuse-type gastric carcinoma (DGC) is characterized by rapid infiltrative growth associated with massive stroma and frequent peritoneal dissemination, which leads to poor patient outcomes. In this study, we found that the oncogenic tyrosine phosphatase SHP2 is tyrosine-phosphorylated downstream of the amplified receptor tyrosine kinases (RTKs) Met and fibroblast growth factor receptor 2 (FGFR2) in DGC cell lines. SHP2 knockdown or pharmacological inhibition selectively suppressed the growth of DGC addicted to amplified Met and FGFR2. Moreover, targeting SHP2 abrogated malignant phenotypes, including peritoneal dissemination, of Met-addicted DGC and could overcome acquired resistance to Met inhibitors. Our findings suggest that SHP2 is a potential target for the treatment of DGC addicted to amplified RTK signaling.

**Abstract:**

Diffuse-type gastric carcinoma (DGC) exhibits aggressive progression associated with rapid infiltrative growth, massive fibrosis, and peritoneal dissemination. Gene amplification of Met and fibroblast growth factor receptor 2 (FGFR2) receptor tyrosine kinases (RTKs) has been observed in DGC. However, the signaling pathways that promote DGC progression downstream of these RTKs remain to be fully elucidated. We previously identified an oncogenic tyrosine phosphatase, SHP2, using phospho-proteomic analysis of DGC cells with Met gene amplification. In this study, we characterized SHP2 in the progression of DGC and assessed the therapeutic potential of targeting SHP2. Although SHP2 was expressed in all gastric carcinoma cell lines examined, its tyrosine phosphorylation preferentially occurred in several DGC cell lines with Met or FGFR2 gene amplification. Met or FGFR inhibitor treatment or knockdown markedly reduced SHP2 tyrosine phosphorylation. Knockdown or pharmacological inhibition of SHP2 selectively suppressed the growth of DGC cells addicted to Met or FGFR2, even when they acquired resistance to Met inhibitors. Moreover, SHP2 knockdown or pharmacological inhibition blocked the migration and invasion of Met-addicted DGC cells in vitro and their peritoneal dissemination in a mouse xenograft model. These results indicate that SHP2 is a critical regulator of the malignant progression of RTK-addicted DGC and may be a therapeutic target.

## 1. Introduction

Gastric cancer is one of the most common malignancy and the leading cause of cancer-related death worldwide [1]. There are two main histological subtypes of gastric carcinoma: intestinal and diffuse [2]. Hallmarks of diffuse-type gastric carcinoma (DGC) include the presence of poorly differentiated carcinoma cells and abundant desmoplastic stroma, rapid infiltration into the submucosa, and high incidence of peritoneal dissemination, a critical determinant for the poor outcome and quality of life [3,4]. Due to these aggressive phenotypes, patients with DGC have a poor prognosis [5,6]. The molecular mechanism underlying the malignant progression of DGC is insufficiently understood; therefore, effective molecular targeted therapy has not yet been established.

Aberrant and oncogenic activation of receptor tyrosine kinases (RTKs) is frequently observed in a diverse range of carcinomas [7]. In the case of DGC, gene amplification of *MET* and *FGFR2* has been reported [8,9,10]. *MET* and *FGFR2* are well-known oncogenes encoding the RTKs Met and fibroblast growth factor receptor 2 (FGFR2), respectively. Met and FGFR2 have been implicated in cancer malignancies, including invasion, metastasis, angiogenesis, and drug resistance [11,12]. Gene amplification of Met and FGFR2 has been correlated with poor prognosis in patients with gastric cancer [8,13,14,15]. Therefore, Met and FGFR2 were proposed as potential therapeutic targets for DGC [16,17]. Indeed, other groups and we demonstrated that Met and FGFR inhibitors have therapeutic efficacy in preclinical models [18,19,20,21,22]; as a result, some of them were tested in clinical trials [23]. However, acquired resistance to Met and FGFR inhibitors inevitably occurs in DGC [22,24], which will be problematic in clinical practice. Moreover, FGFR2 overexpression gives rise to inherent resistance to Met inhibitors in DGC with Met gene amplification [25]. This reinforced a critical need to identify signaling proteins that act as nodes relaying oncogenic signals downstream of multiple RTKs, which could be therapeutic targets.

SHP2 is an oncogenic non-receptor protein tyrosine phosphatase that functions in cell signaling [26]. Germ line mutations in the SHP2 gene result in Noonan syndrome and Leopard syndrome, both of which are predisposed to an increased risk of developing tumors [27,28]. Recurrent somatic mutations in the phosphatase have been found in diverse types of cancers [27,28,29,30,31]. SHP2 is tyrosine-phosphorylated at Y542 and Y580 downstream of RTKs, which is thought to activate SHP2 molecules by releasing them from an autoinhibitory conformation [32,33]. Cellular functions of SHP2 include regulation of cell growth, survival, and migration, mainly via Ras/ERK pathway activation [26,28]. Thus, SHP2 has emerged as a promising therapeutic target for cancer [29]. Indeed, several SHP2 allosteric inhibitors have been developed and shown to exhibit therapeutic potential for RTK-, Ras-, BRAF-, and NF1-driven cancers in preclinical models [34,35,36,37,38]. However, the importance of SHP2 and the therapeutic efficacy of targeting SHP2 in DGC remain to be determined.

We previously conducted phospho-proteomic analysis of DGC cells with Met gene amplification and identified several proteins that are tyrosine phosphorylated downstream of Met [21,39]. Here, we investigated the role of SHP2 in the progression of DGC. We found that SHP2 plays a key role in the growth and peritoneal dissemination of DGC addicted to RTK signaling.

## 2. Materials and Methods

### 2.1. Cell Culture

The human gastric cancer cell lines, HSC-39, HSC-43, HSC-59, HSC-60, HSC-64, HSC-44PE, 58As9, 58As1, and 44As3, have been described previously [40,41,42,43,44]. The human gastric cancer cell lines, MKN1, MKN7, MKN74, NUGC-4, KATO-III, MKN45, IM95, and human lung cancer cell line EBC-1 were obtained from the JCRB Cell Bank (Osaka, Japan). The human gastric cancer cell lines, NCI-N87 and SNU-5, human lung cancer cell line A549, and human mesothelial cell line Met5A were obtained from ATCC (Rockville, MD, USA). The human gastric cancer cell lines, GCIY, ECC12, H-111-TC, GSU, and KE-97, were provided by the RIKEN BRC through the National Bio-Resource Project of the MEXT, Japan (Ibaraki, Japan). These cells were maintained in RPMI 1640 medium (Thermo Fisher Scientific, Waltham, MA, USA) supplemented with 10% fetal bovine serum, 10 U/mL of penicillin, and 10 µg/mL of streptomycin at 37 °C in a humidified atmosphere containing 5% CO_2_. Mycoplasma contamination was tested using a MycoAlert Mycoplasma Detection Kit (Lonza, Basel, Switzerland).

### 2.2. Antibodies and Reagents

SHP2, Met, ERK, Akt, and β-actin antibodies, including phospho-specific ones, were purchased from Cell Signaling Technology (Danvers, MA, USA). Anti-phospho-SHP2 Y542 antibody was purchased from Abcam (Cambridge, UK). FGFR2α and phospho-FGFR1-4 antibodies were purchased from R&D Systems (Minneapolis, MN, USA). PHA-665752 and JNJ-38877605 were obtained from Selleck Chemicals (Houston, TX, USA). PD-173074 and SHP099 were purchased from Sigma–Aldrich (St. Louis, MO, USA) and Cayman Chemical (Ann Arbor, MI, USA), respectively.

### 2.3. siRNA Transfection

Stealth RNAi molecules against SHP2 (#1, HSS108834; #2, HSS184068; #3, HSS184069) and negative control were purchased from Thermo Fisher Scientific. Cells were transfected with the indicated siRNAs using Lipofectamine RNAiMAX Reagent (Thermo Fisher Scientific) in accordance with the manufacturer’s instructions. The cells were cultured for 24–72 h and subjected to immunoblotting and other assays. For the cell growth assay, cells were transfected with siRNA again at 3 days after the first transfection and cultured for another 3 days before the assay.

### 2.4. Lentiviral shRNA Transduction

Lentiviral shRNA constructs were generated by ligating oligo DNA for control shRNA (CCGGCCTAAGGTTAAGTCGCCCTCGCTCGAGCGAGGGCGACTTAACCTTAGGTTTTT) or SHP2 shRNA (CCGGGGAAAGGGCACGAATATACAACTCGAGTTGTATATTCGTGCCCTTTCCTTTTT) into pLKO.1-puro vector (a generous gift from Bob Weinberg (Whitehead Institute, MIT, Cambridge, MA, USA) [45], plasmid #8453, Addgene, Watertown, MA, USA). Lentiviruses were generated with the pLKO.1-puro constructs and ViraPower (Thermo Fisher Scientific) or pMD2.G (Addgene plasmid #12259)/psPAX2 (Addgene plasmid #12260) in 293FT (Thermo Fisher Scientific) or Lenti-X 293T cells (Takara, Shiga, Japan). Two to three days after transfection, the cell supernatant containing lentiviral particles was collected. The 58As9 cells were infected with the lentiviruses for 1 d and then subjected to intraperitoneal injection into nude mice 2 days after infection.

### 2.5. Immunoblotting

Cells were washed twice with ice-cold PBS before lysis in a buffer containing 50 mM of HEPES-NaOH (pH 7.0), 150 mM of NaCl, 10% glycerol, 1% Triton X-100, 1.5 mM of MgCl_2_, 1 mM of EGTA, 1 mM of Na_3_VO_4_, and a protease inhibitor cocktail (Roche, Rotkreuz, Switzerland). Protein concentration was determined using a BCA protein assay kit (Thermo Fisher Scientific). The samples were resolved using SDS-PAGE, transferred to PVDF membranes, and blocked with Blocking One (Nacalai Tesque, Kyoto, Japan). The membranes were incubated first with primary antibodies for 1 h and then with peroxidase-conjugated secondary antibodies for 30 min. Immunoreactive bands were detected using Pierce ECL Plus Western Blotting Substrate (Thermo Fisher Scientific). Whole Western Blots can be found in File S1.

### 2.6. Cell Proliferation Assay

Cells seeded onto 96-well plates at 1–2 × 10^3^ cells/well were treated with siRNAs or inhibitors and cultured. Premix WST-1 Cell Proliferation Assay System (Takara) or Cell Counting Kit-8 (Dojindo, Kumamoto, Japan) were used to determine cell viability in accordance with to the manufacturer’s instructions.

### 2.7. Quantitative PCR (qPCR)

Total RNA was isolated using the RNeasy Plus Mini Kit (Qiagen, Venlo, The Netherlands). Template cDNA was synthesized using ReverTra Ace (Toyobo, Osaka, Japan). qPCR was performed using Thunderbird qPCR Mix (Toyobo) in a CFX96 Real-Time PCR Detection System (Bio-Rad Laboratories, Hercules, CA, USA). The sequences of the primer pairs used were as follows: GAPDH Forward, GTGAAGGTCGGAGTCAACG; GAPDH Reverse, TGAGGTCAATGAAGGGGTC; SHP2 Forward, ACGGCAAGTCTAAAGTGACC; SHP2 Reverse, ACTGTACCCAATGTTTCCACC.

### 2.8. Cell Migration and Invasion Assays

FluoroBlok 24-multiwell insert systems (8-μm pore size, Corning, Corning, NY, USA) were employed to determine cell migration and invasion. Matrigel (1 mg/mL, 30 µL) was added to the inserts and solidified for 1 h at 37 °C for invasion assay. For fluorescent labeling, 58As9 cells were incubated with 20 µM of calcein-AM (Dojindo) for 30 min at 37 °C. The labeled cells (1 × 10^5^) suspended in 300 µL of serum-free medium were added to the upper chambers. As a chemoattractant, 800 μL of growth medium containing serum was added to the lower chambers. The cells were cultured for 16 h for migration or 24 h for invasion. Subsequently, the migrated/invaded cells were directly imaged by fluorescent microscopy and quantified in five randomly selected fields per insert.

### 2.9. Peritoneal Dissemination Assay

58As9 cells (2 × 10^6^) were inoculated intraperitoneally into BALB/c nude mice (6-week-old, female) purchased from CLEA Japan (Tokyo, Japan). Fifteen days after inoculation, the mice were euthanized and dissected to evaluate peritoneal dissemination and ascites formation. For the inhibitor experiments, the mice were intraperitoneally administered DMSO or SHP099 at 30 mg/kg thrice a week.

### 2.10. Statistical Analysis

Data are representative of at least three independent experiments. Statistical analysis was performed using GraphPad Prism 8 (GraphPad Software, San Diego, CA, USA). Statistical significance (defined as *p* < 0.05) was calculated using a two-tailed Student’s *t*-test with Welch’s correction, one-way ANOVA, and Tukey’s multiple comparison test, or Mann–Whitney U test. *p*-values: ns = not significant, *p* > 0.05; *, *p* < 0.05; **, *p* < 0.01; ***, *p* < 0.001; ****, *p* < 0.0001.

## 3. Results

### 3.1. SHP2 Is Preferentially Tyrosine Phosphorylated in DGC Cell Lines with Met or FGFR2 Gene Amplification

**Figure 1:**
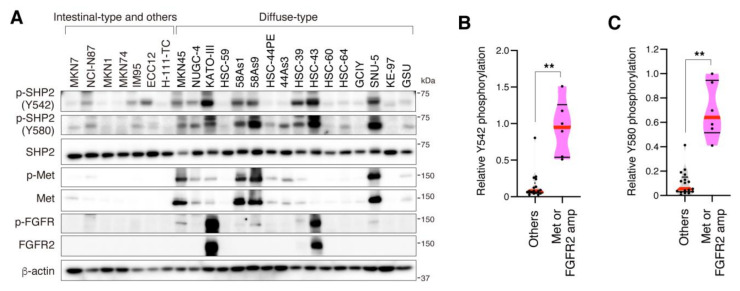
Tyrosine phosphorylation of SHP2 in gastric carcinoma cell lines. (**A**) Human gastric carcinoma cell lines were subjected to immunoblotting with indicated antibodies. (**B**,**C**) Violin plots show relative phosphorylation levels of SHP2 Y542 (**B**) and Y580 (**C**) that were calculated from the immunoblot data and compared between cell lines with or without Met or FGFR2 gene amplification. Red lines denote medians and black lines denote quartiles (*n* = 17 for others and 6 for Met or FGFR2 gene amplification). **, *p* < 0.01.

Phosphorylation and expression of SHP2 were first examined in a panel of human gastric cancer cell lines by immunoblot analysis (Figure 1A). SHP2 expression was detected in all the cell lines examined. In contrast, phosphorylation of SHP2 at Y542 and Y580 was preferentially detected in several DGC cell lines with gene amplification of RTKs: MKN45, 58As1, 58As9, and SNU-5, which have Met gene amplification [46,47]; KATO-III and HSC-43, which have FGFR2 gene amplification [48]. Quantitative analyses confirmed that the phosphorylation of SHP2 at Y542 and Y580 was significantly higher in cell lines with Met or FGFR2 gene amplification than in other cell lines (Figure 1B,C). Strong phosphorylation of SHP2 at Y542 and Y580 was also observed in the lung cancer cell line EBC-1, which has Met gene amplification, whereas SHP2 was significantly less phosphorylated in the lung cancer cell line A549 and normal mesothelial cell line Met5A (Figure S1A,B).

**Figure 2:**
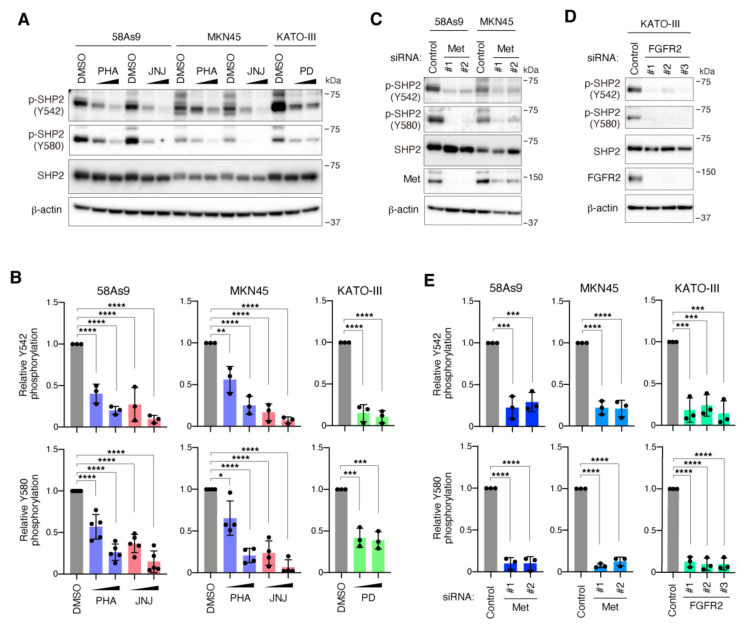
SHP2 is tyrosine-phosphorylated downstream of amplified Met and FGFR2 in DGC cells. (**A**) Immunoblot analysis of 58As9 and MKN45 cells treated with Met inhibitors PHA-665752 (PHA) and JNJ-38877605 (JNJ), and of KATO-III cells treated with the FGFR inhibitor PD-173074 (PD), at 100 or 300 nM for 2 h. (**B**) Quantitative analysis of immunoblot data for SHP2 phosphorylation in cells treated with inhibitors. Bars, SD (*n* = 5 for 58As9 Y580, 4 for MKN45 Y580, and 3 for others). *, *p* < 0.05; **, *p* < 0.01; ***, *p* < 0.001; ****, *p* < 0.0001. (**C**) 58As9 and MKN45 cells were transfected with control or Met siRNAs and subjected to immunoblot analysis. (**D**) KATO-III cells were transfected with control or FGFR2 siRNAs and subjected to immunoblot analysis. (**E**) Quantification of SHP2 phosphorylation in cells transfected with siRNAs. Bars, SD (*n* = 3). ***, *p* < 0.001; ****, *p* < 0.0001.

Treatment of 58As9 and MKN45 cells with Met inhibitors, PHA-665752 and JNJ-38877605, markedly decreased the tyrosine phosphorylation of SHP2 at both Y542 and Y580 in a dose-dependent manner (Figure 2A,B). Likewise, treatment of KATO-III cells with the FGFR inhibitor PD-173074 reduced SHP2 phosphorylation (Figure 2A,B). Treatment of 58As9 and MKN45 cells with PD-173074 or KATO-III cells with PHA-665752 and JNJ-38877605 did not obviously affect SHP2 phosphorylation, confirming the specificity of these inhibitors (Figure S2). Met knockdown with two different siRNAs also decreased SHP2 phosphorylation in both 58As9 and MKN45 cells (Figure 2C,E). SHP2 phosphorylation was also significantly reduced by FGFR2 silencing with three different siRNAs in KATO-III cells (Figure 2D,E). These results indicate that the phosphorylation of SHP2 at Y542 and Y580 is dependent on oncogenic signals of amplified Met and FGFR2 in DGC.

### 3.2. SHP2 Is Required for the Growth of DGC Cells with Met or FGFR2 Gene Amplification

**Figure 3:**
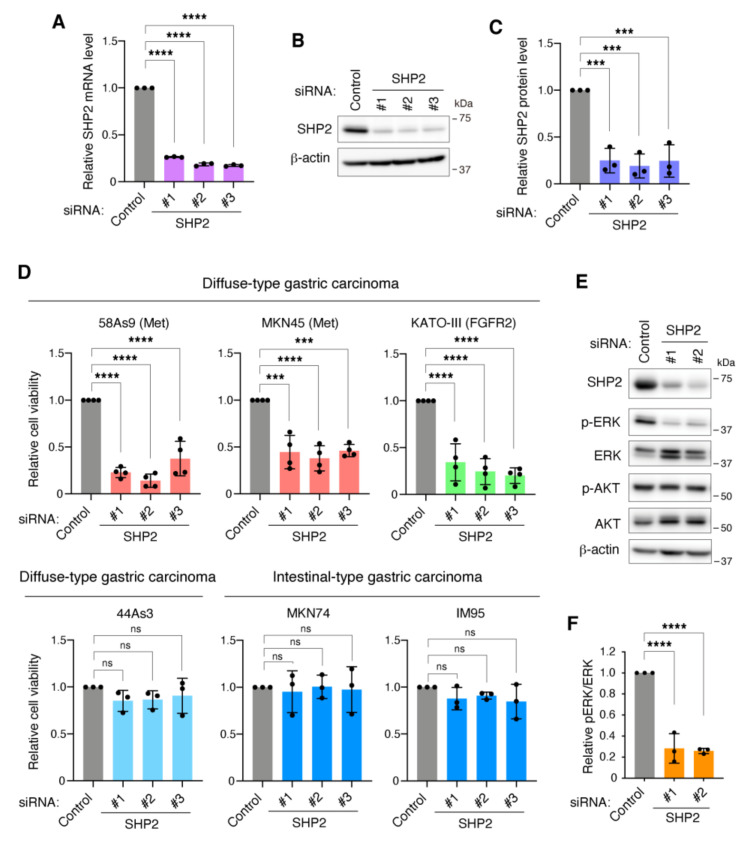
SHP2 is required for the growth of DGC cells addicted to Met or FGFR2. (**A**) 58As9 cells transfected with control or SHP2 siRNAs were subjected to qPCR analysis. Bars, SD (*n* = 3). ****, *p* < 0.0001. (**B**) 58As9 cells transfected with siRNAs were subjected to immunoblot analysis. (**C**) Quantitative analysis of immunoblot data for SHP2 protein levels. Bars, SD (*n* = 3). ***, *p* < 0.001. (**D**) Gastric carcinoma cells were transfected with control or SHP2 siRNAs for 6 days and examined for their viability. Bars, SD (*n* = 4 for 58As9, MKN45, KATO-III and 3 for others). ***, *p* < 0.001; ****, *p* < 0.0001, ns = not significant. (**E**) 58As9 cells transfected with siRNAs were subjected to immunoblot analysis. (**F**) Quantitative analysis of ERK phosphorylation. Bars, SD (*n* = 3). ****, *p* < 0.0001.

Next, we examined the requirement of SHP2 for the growth of gastric carcinoma cells. Transfection of 58As9 cells with three different siRNAs against SHP2 resulted in a marked reduction of SHP2 at both the mRNA and protein levels (Figure 3A–C). SHP2 knockdown severely impaired the growth of 58As9 cells with Met gene amplification (Figure 3D). Similar results were obtained in MKN45 and KATO-III cells with Met and FGFR2 gene amplification, respectively. In contrast, SHP2 silencing did not affect the growth of other intestinal-type and DGC cells, MKN74, IM95, and 44As3. Efficacy of SHP2 knockdown in these cell lines was confirmed by immunoblotting (Figure S3). SHP2 knockdown also reduced the growth of EBC-1 lung carcinoma cells with Met gene amplification, but not in Met5A normal mesothelial cells (Figure S1C). As expected, SHP2 knockdown significantly reduced the phosphorylation of ERK, but not of Akt, in 58As9 cells (Figure 3E,F). Taken together, these observations indicate that SHP2 is required for the growth of DGC cells with gene amplification of Met or FGFR2.

### 3.3. SHP2 Knockdown Blocks Migration, Invasion, and Peritoneal Dissemination of Met-Addicted DGC Cells

**Figure 4:**
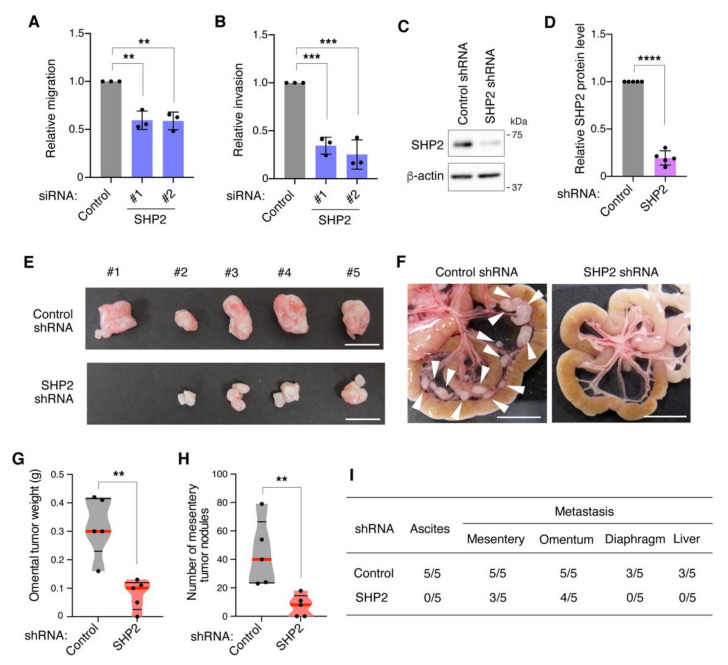
SHP2 is required for malignant phenotypes of Met-addicted DGC cells. (**A**,**B**) 58As9 cells were transfected with control or SHP2 siRNAs for 1 day and examined for their migration (**A**) and invasion (**B**) by Transwell assays. Bars, SD (*n* = 3). **, *p* < 0.01, ***, *p* < 0.001. (**C**) 58As9 cells expressing control or SHP2 shRNA were subjected to immunoblot analysis. (**D**) SHP2 expression was quantitated with immunoblot data. Bars, SD (*n* = 5). ****, *p* < 0.0001. (**E**,**F**) 58As9 cells expressing control or SHP2 shRNA were intraperitoneally injected into nude mice. Macroscopic images of omental tumors (**E**) and mesentery (**F**) at 15 days after injection are shown. Scale bars, 1 cm. Arrowheads denote mesentery tumors. (**G**,**H**) Omental tumor weight (**G**) and the number of mesentery tumors equal to or larger than 1 mm in diameter (**H**) are shown in violin plots. Red lines denote medians and black lines denote quartiles (*n* = 5). **, *p* < 0.01. (**I**) Number of mice bearing ascites or tumors at indicated site per total number of mice injected.

The role of SHP2 in the malignant phenotypes of DGC, cell migration, and invasion, was then examined in vitro. To exclude a possibility that the suppression of cell growth by SHP2 silencing affects cell migration and invasion, the assays were carried out during 1–2 days after siRNA transfection when cell viability was not significantly affected (Figure S4A). SHP2 silencing in 58As9 cells blocked their ability to migrate and invade the reconstituted basement membrane in Transwell assays (Figure 4A,B). We then assessed the effect of targeting SHP2 on peritoneal dissemination using a mouse xenograft model. 58As9 cells were infected with lentiviruses expressing control shRNA or SHP2 shRNA. Reduction of SHP2 protein levels was confirmed by immunoblot analysis (Figure 4C,D). The cells were intraperitoneally inoculated into nude mice, and their ability to form tumors in the abdominal tissues was evaluated. SHP2 knockdown cells exhibited a diminished ability to form omental tumors compared with control cells (Figure 4E,G). In addition, the number of mesentery tumors markedly decreased in SHP2 knockdown cells (Figure 4F,H). The frequency of ascites formation and metastasis to the diaphragm and liver was also reduced by SHP2 knockdown (Figure 4I). These results demonstrate that SHP2 is necessary for malignant phenotypes, including migration, invasion, and peritoneal dissemination, in DGC cells with Met gene amplification.

### 3.4. Pharmacological Inhibition of SHP2 Abrogates Malignant Phenotypes of Met-Addicted DGC Cells

**Figure 5:**
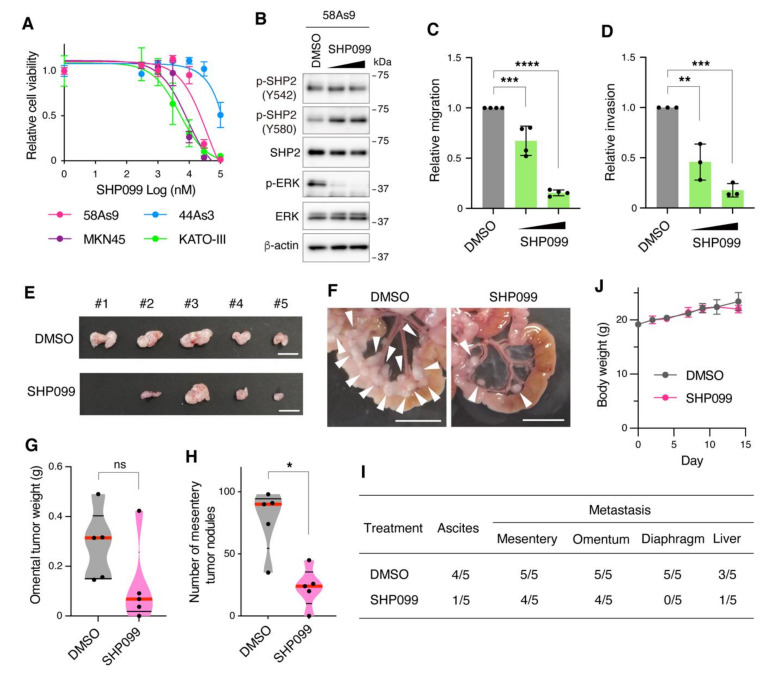
Pharmacological inhibition of SHP2 abrogates the growth and malignant phenotypes of Met-addicted DGC cells. (**A**) 58As9, MKN45, KATO-III, and 44As3 cells were treated with increasing concentrations of the allosteric SHP2 inhibitor SHP099 for 6 days and their viability was examined. Bars, SD (*n* = 3). (**B**) 58As9 cells were treated with DMSO or SHP099 (10 or 30 µM) for 2 h and subjected to immunoblotting. (**C**,**D**) 58As9 cells were examined for their migration (**C**) and invasion (**D**) in the presence of DMSO or SHP099 (30 or 100 µM) by Transwell assays. Bars, SD (*n* = 3). **, *p* < 0.01; ***, *p* < 0.001; ****, *p* < 0.0001. (**E**,**F**) 58As9 cells were intraperitoneally injected into nude mice. The mice were intraperitoneally administered DMSO or SHP099 at 30 mg/kg thrice a week. Macroscopic images of omental tumors (**E**) and mesentery (**F**) at 15 days after injection are shown. Scale bars, 1 cm. Arrowheads denote mesentery tumors. (**G**,**H**) Omental tumor weight (**G**) and the number of mesentery tumors equal to or larger than 1 mm in diameter (**H**) are shown in violin plots. Red lines denote medians and black lines denote quartiles (*n* = 5). *, *p* < 0.05, ns = not significant. (**I**) Number of mice bearing ascites or tumors at indicated site per total number of mice injected. (**J**) Body weight of the mice. Bars, SD (*n* = 5).

The effect of pharmacological SHP2 inhibition on the growth of DGC cells was examined. Treatment of cells with SHP099, a specific SHP2 allosteric inhibitor, preferentially suppressed the growth of DGC cells with Met or FGFR2 gene amplification, 58As9, MKN45, and KATO-III, in a dose-dependent manner (Figure 5A). In contrast, 44As3 cells without RTK amplification were less sensitive to SHP099. SHP099 treatment drastically reduced ERK phosphorylation without affecting SHP2 phosphorylation in 58As9 cells (Figure 5B). Next, the effect of SHP099 on the malignant phenotypes of DGC cells was assessed in vitro and in vivo. Treatment of 58As9 cells with SHP099 significantly suppressed cell migration and invasion in Transwell assays (Figure 5C,D). The assays were carried out within 1 day after SHP099 treatment when cell viability was only slightly affected (Figure S4B). The therapeutic efficacy of SHP099 for peritoneal dissemination of DGC was tested in a mouse xenograft model. Intraperitoneal administration of SHP099 reduced the formation of omental tumors by 58As9 cells, although the difference was not statistically significant (Figure 5E,G). In contrast, SHP099 administration significantly reduced the number of mesentery tumors (Figure 5F,H). Moreover, SHP099 administration reduced the incidence of ascites formation and metastasis to the liver and diaphragm (Figure 5I). No obvious changes in body weight or overall health status were observed over the course of the experiment (Figure 5J). Thus, these results suggest that pharmacological inhibition of SHP2 is effective in blocking the growth and malignant phenotypes of Met-addicted DGC cells.

### 3.5. Inhibition of SHP2 Overcomes Resistance to Met Inhibitors in DGC Cells

**Figure 6:**
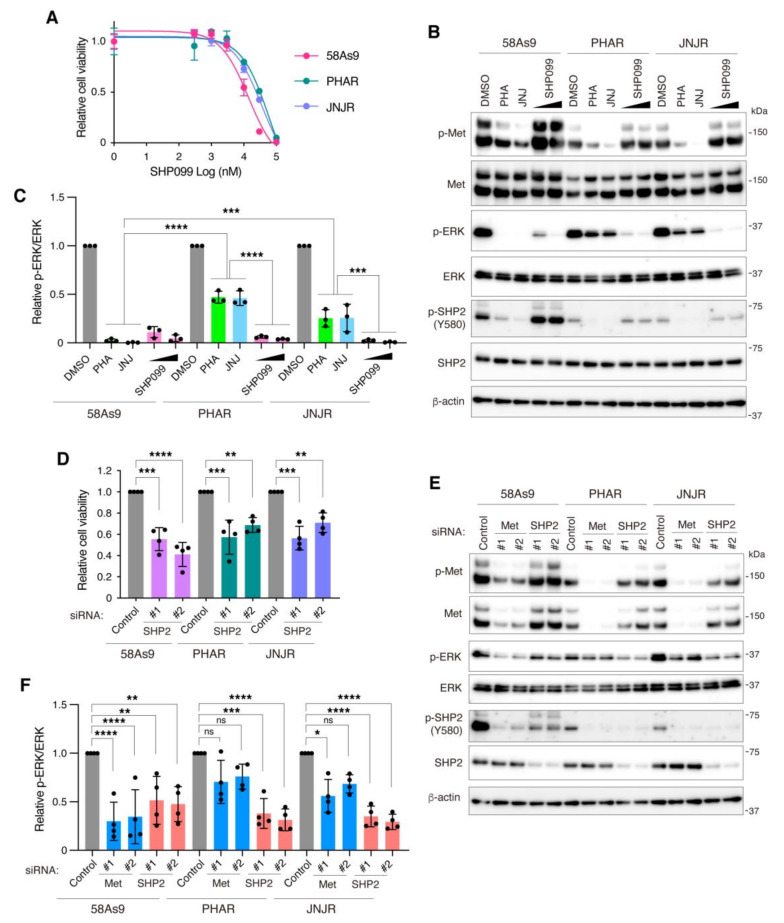
Blocking SHP2 overcomes resistance to Met inhibitors in DGC cells. (**A**) Parental 58As9 and Met inhibitor resistant sublines, PHAR and JNJR cells, were treated with increasing concentrations of the allosteric SHP2 inhibitor SHP099 for 6 days and the cell viability was examined. Bars, SD (*n* = 3). (**B**) 58As9, PHAR, and JNJR cells were treated with Met inhibitors, PHA-665752 (PHA) and JNJ-38877605 (JNJ) at 300 nM, or SHP099 at 10 or 30 µM for 2 h and subjected to immunoblot analysis. (**C**) Relative levels of ERK phosphorylation were determined by quantitative analysis of the immunoblotting data. Bars, SD (*n* = 3). ***, *p* < 0.001; ****, *p* < 0.0001. (**D**) 58As9, PHAR, and JNJR cells were transfected with control or SHP2 siRNAs and their viability was examined at 6 days after transfection. Bars, SD (*n* = 4). **, *p* < 0.01, ***, *p* < 0.001; ****, *p* < 0.0001. (**E**) 58As9, PHAR, and JNJR cells transfected with siRNAs were subjected to immunoblot analysis. (**F**) Quantitative analysis of ERK phosphorylation. Bars, SD (*n* = 4). *, *p* < 0.05; **, *p* < 0.01; ***, *p* < 0.001; ****, *p* < 0.0001, ns = not significant.

Finally, we tested whether blockage of SHP2 can overcome acquired resistance to Met inhibitors in DGC cells. We previously established 58As9 cells with acquired resistance to Met inhibitors, namely PHAR and JNJR, the growth of which became insensitive to Met inhibitor treatment and knockdown [39]. Treatment of these cells with SHP099 successfully reduced cell viability to a similar extent as the parental 58As9 cells (Figure 6A). Met inhibitor treatment markedly reduced Met and SHP2 phosphorylation in PHAR and JNJR cells, although a significant amount of phosphorylated ERK remained, as compared to parental 58As9 cells (Figure 6B,C). In contrast, SHP099 treatment nearly completely inhibited ERK phosphorylation in PHAR and JNJR cells, similar to 58As9 cells, without affecting Met and SHP2 phosphorylation. SHP2 knockdown by siRNAs also suppressed the growth of PHAR and JNJR cells (Figure 6D) and markedly suppressed ERK phosphorylation in PHAR and JNJR cells, whereas Met knockdown insignificantly or only slightly reduced it (Figure 6E,F). These findings demonstrate that the growth of DGC cells that acquired resistance to Met inhibitors still depends on SHP2.

## 4. Discussion

Tyrosine phosphorylation of SHP2 at Y452 and Y580 occurs downstream of RTK signaling [32,33]. Gene amplification of RTK causes overexpression and clustering of RTK, resulting in constitutive kinase activation and oncogenic signals [7]. Consistently, we observed that Y452 and Y580 of SHP2 were highly phosphorylated in DGC cell lines with Met or FGFR2 gene amplification, and the phosphorylation was dependent on Met or FGFR2 activity. As we previously reported, these DGC cell lines were addicted to Met or FGFR2, and therefore highly sensitive to Met or FGFR inhibitors [21]. In addition, we demonstrated in this study that DGC cell lines with high levels of SHP2 phosphorylation were sensitive to SHP2 silencing and pharmacological inhibition. These observations imply that Y542 and Y580 phosphorylation of SHP2 can serve as a biomarker to identify patients with tumors that harbor RTK gene amplification and are dependent on RTK signaling and SHP2 for growth. These patients may benefit from the use of RTK and SHP2 inhibitors, either alone or in combination. A recent study showed that SHP2 phosphorylation status, but not expression level, is closely correlated with poor prognosis in breast cancer patients [49]. Although SHP2 was reported to be upregulated in gastric cancer [50], its phosphorylation status has not yet been examined. Further clinicopathological studies are required to elucidate the correlation between SHP2 phosphorylation and gene amplification of RTK, as well as the survival of patients with DGC.

We showed that SHP2 knockdown suppressed the growth of not only DGC cells but also lung carcinoma cells addicted to Met. This is consistent with previous studies demonstrating the requirement of SHP2 for the growth of a variety of cancers driven by RTK signaling [34]. In contrast, SHP2 silencing barely affected the growth of other carcinomas and normal mesothelial cells. The MEK/ERK pathway is more broadly activated by various upstream signaling besides RTK activation. Therefore, sustained shutdown of the MEK/ERK pathway in normal tissue may not be tolerated well [51]. Thus, targeting SHP2 may be a superior therapeutic approach than directly targeting MEK/ERK pathway, against a wide variety of tumors with Met or FGFR2 gene amplification with minimal side effects.

Peritoneal dissemination is the most critical factor that influences patient outcomes in DGC [3]. We showed that knockdown or pharmacological inhibition of SHP2 in DGC cells blocked cell migration and invasion in vitro and abrogated peritoneal dissemination in a mouse xenograft model. Although the involvement of SHP2 in hematogenous metastasis has been reported in several cancer types [52,53,54], this is the first study to demonstrate the importance of SHP2 in peritoneal dissemination. DGC cells dispersed in the abdominal cavity must attach to and migrate across the mesothelium, invade the underlying stroma, and regrow to form peritoneal tumors [23,55,56]. Based on our results, SHP2 is likely to participate in the migration, invasion, and growth of DGC cells during peritoneal dissemination. Our findings highlight a promising approach for targeting SHP2 for the treatment of peritoneal dissemination of DGC.

Pharmacological targeting of SHP2 has emerged as an attractive therapeutic option for the treatment of several cancers [29]. Here, we found that the allosteric SHP2 inhibitor SHP099 potently suppressed the growth and peritoneal dissemination of DGC cells with Met gene amplification. In addition to its role in cancer cells, SHP2 is a critical effector for immune checkpoint signaling in cytolytic T cells, and SHP2 inhibition triggers anti-tumor immunity [57]. Therefore, SHP2 inhibitor treatment may have better anti-tumorigenic effects than RTK inhibitor treatment, not only by blocking RTK signaling in tumor cells but also by enhancing anti-tumor immunity. To evaluate this idea, immunocompetent, such as syngeneic, mouse models must be used in future studies.

Acquired drug resistance is a major issue in targeted cancer therapy. The molecular mechanisms underlying resistance to RTK inhibitor include resistance mutations in RTK and activation of alternate RTKs [7]. In addition, adaptive resistance to MEK/ERK pathway inhibitors often occurs via RTK activation [58,59]. Considering that SHP2 functions downstream of multiple RTKs, targeting SHP2 should be effective in a large proportion of tumors with resistance to RTK and MEK/ERK pathway inhibitors. Indeed, several studies have demonstrated that SHP2 inhibition can overcome resistance to EGFR, HER2, ALK, and MEK inhibitors in multiple cancer models [49,60,61,62]. In DGC, acquired resistance to Met and FGFR inhibitors has been reported [22,24,25]. Here, we revealed that SHP2 inhibition can successfully block ERK activity and cell growth in DGC cells with acquired resistance to Met inhibitors. Our results are supported by a recent report showing that SHP2 inhibition abrogates the growth of lung cancer cells with Met inhibitor resistance [63]. Thus, the use of SHP2 inhibitors may be a critical alternative strategy for the treatment of DGC that acquired resistance to RTK inhibitors.

## 5. Conclusions

In conclusion, our results demonstrated that targeting SHP2 is an effective approach for blocking the malignant progression of DGC addicted to amplified RTKs, at least in preclinical models. Our findings provide a strong rationale for the clinical evaluation of SHP2 inhibitors in patients with DGC with RTK gene amplification.

## Data Availability

Data and information are included in the article and Supplementary Materials are available from the authors upon reasonable request.

## Supplementary Materials

The following are available online at https://www.mdpi.com/article/10.3390/cancers13174309/s1, Figure S1: SHP2 phosphorylation and knockdown in lung cancer and mesothelial cell lines, Figure S2: Immunoblot analysis of SHP2 phosphorylation in gastric carcinoma cells treated with inhibitors, Figure S3: Immunoblot analysis of SHP2 expression in gastric carcinoma cells transfected with control or SHP2 siRNAs, Figure S4: Time-course of cell viability after SHP2 siRNA transfection or inhibitor treatment. File S1: Whole Western Blots.
