# Supplementary material for: SHP2 as a Potential Therapeutic Target in Diffuse-Type Gastric Carcinoma Addicted to Receptor Tyrosine Kinase Signaling
Source: Cancers (Basel). 2021 Aug 26;13(17):4309. doi: 10.3390/cancers13174309 (PMC8430696; doi:10.3390/cancers13174309)

Full immunoblot data

Full western blots for Figure 1A

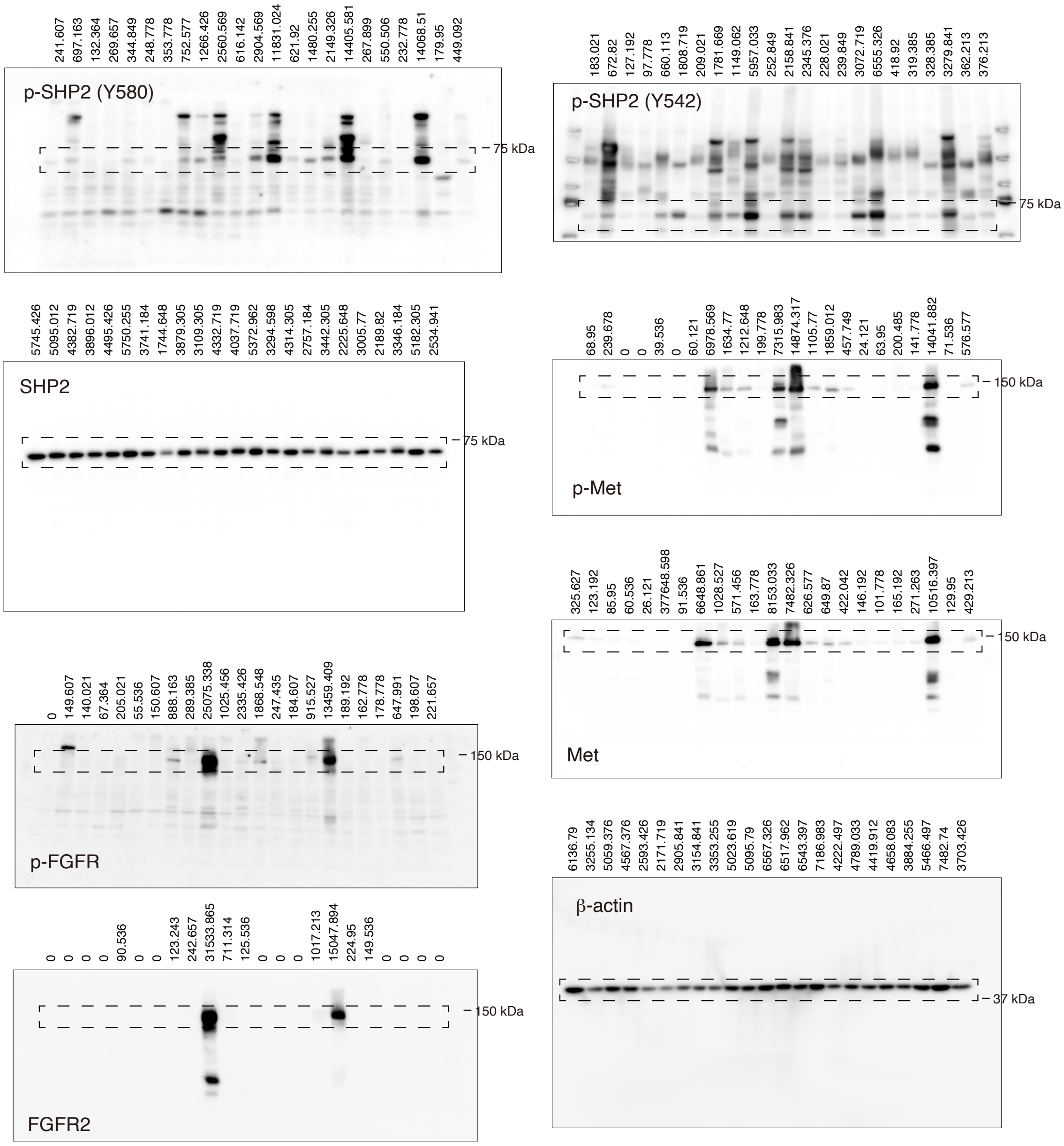

Full western blots for Figure 2A

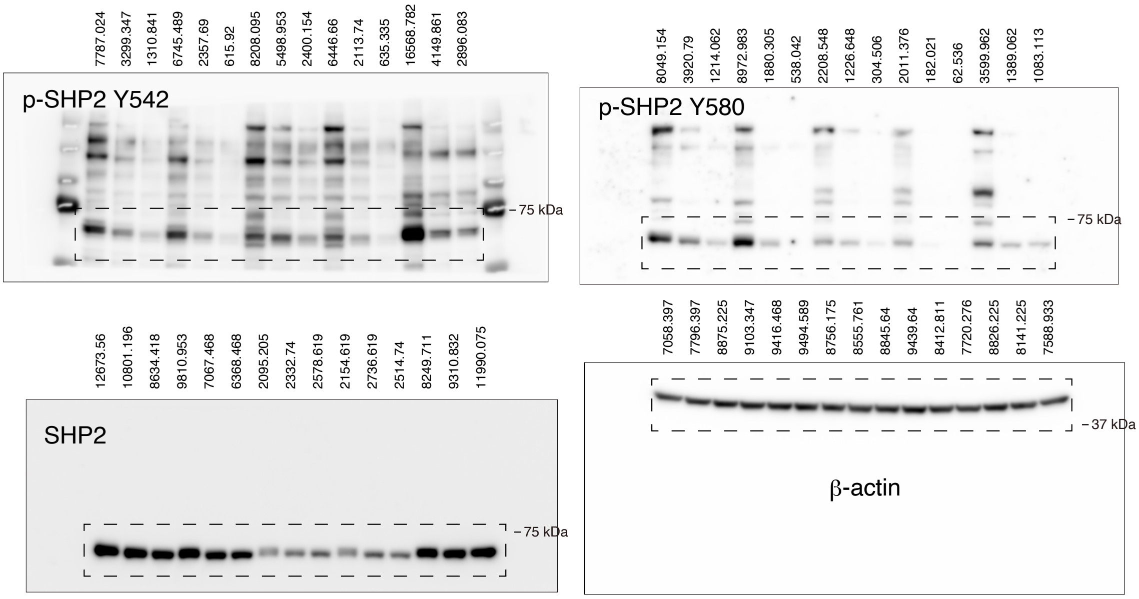

Full western blots for Figure 2C

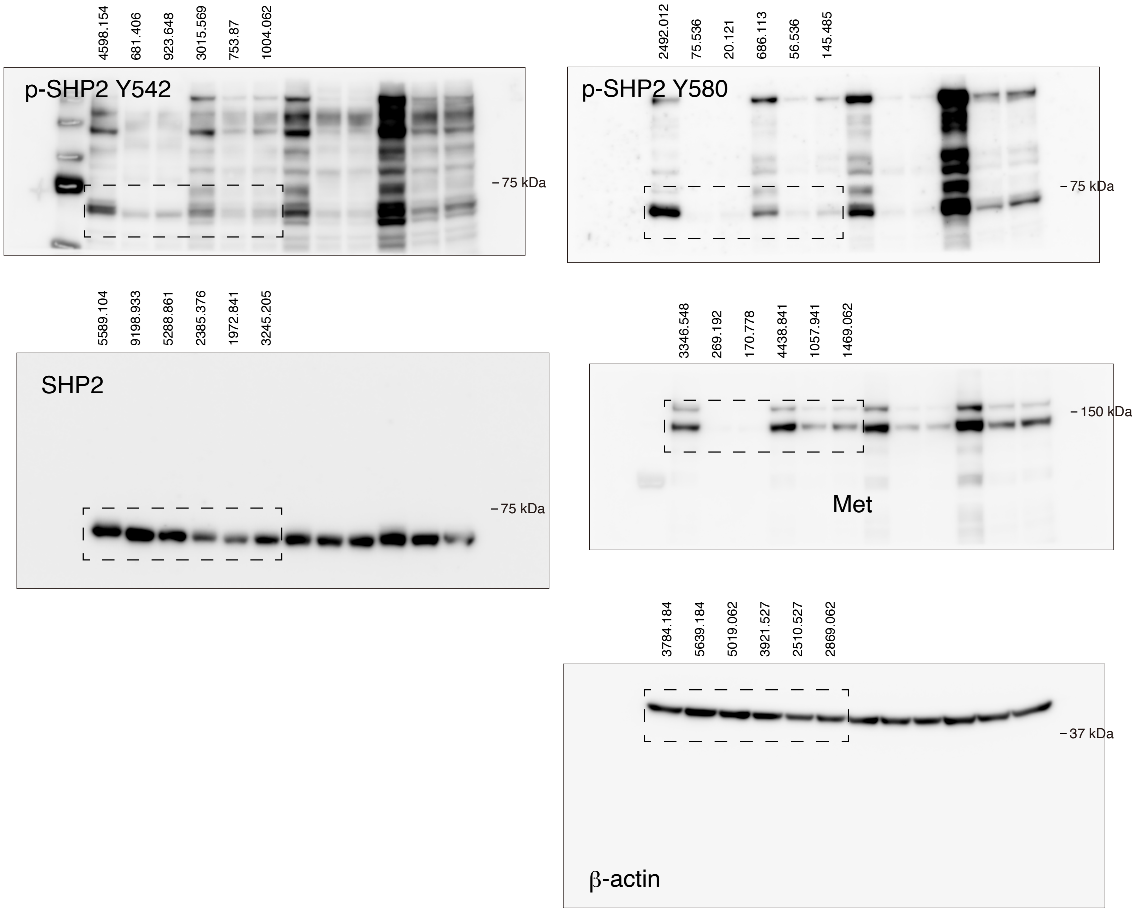

Full western blots for Figure 2D

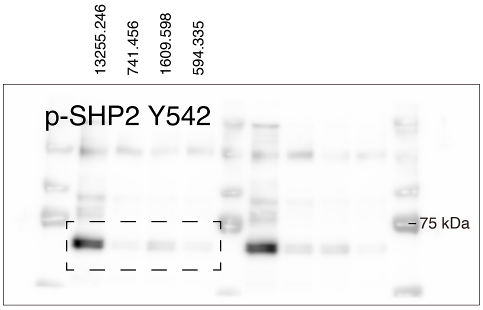

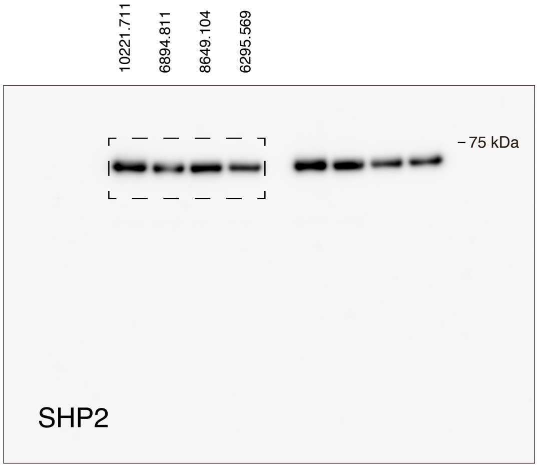

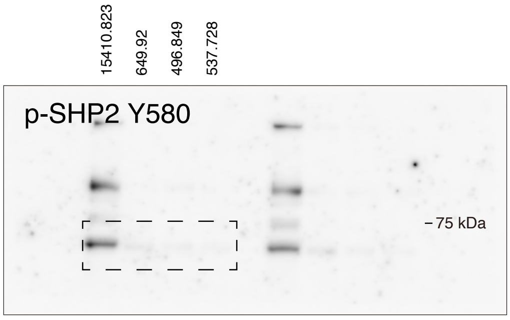

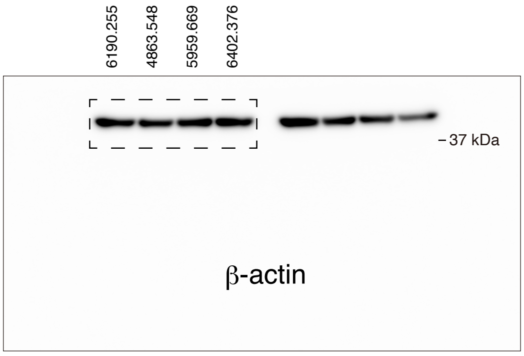

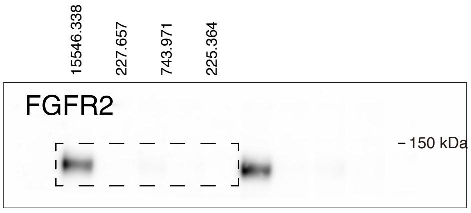

Full western blots for Figure 3B

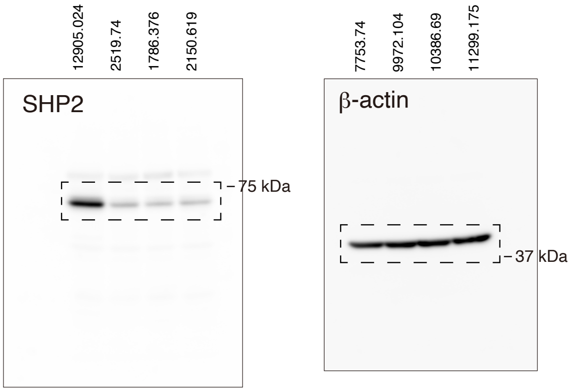

Full western blots for Figure 3E

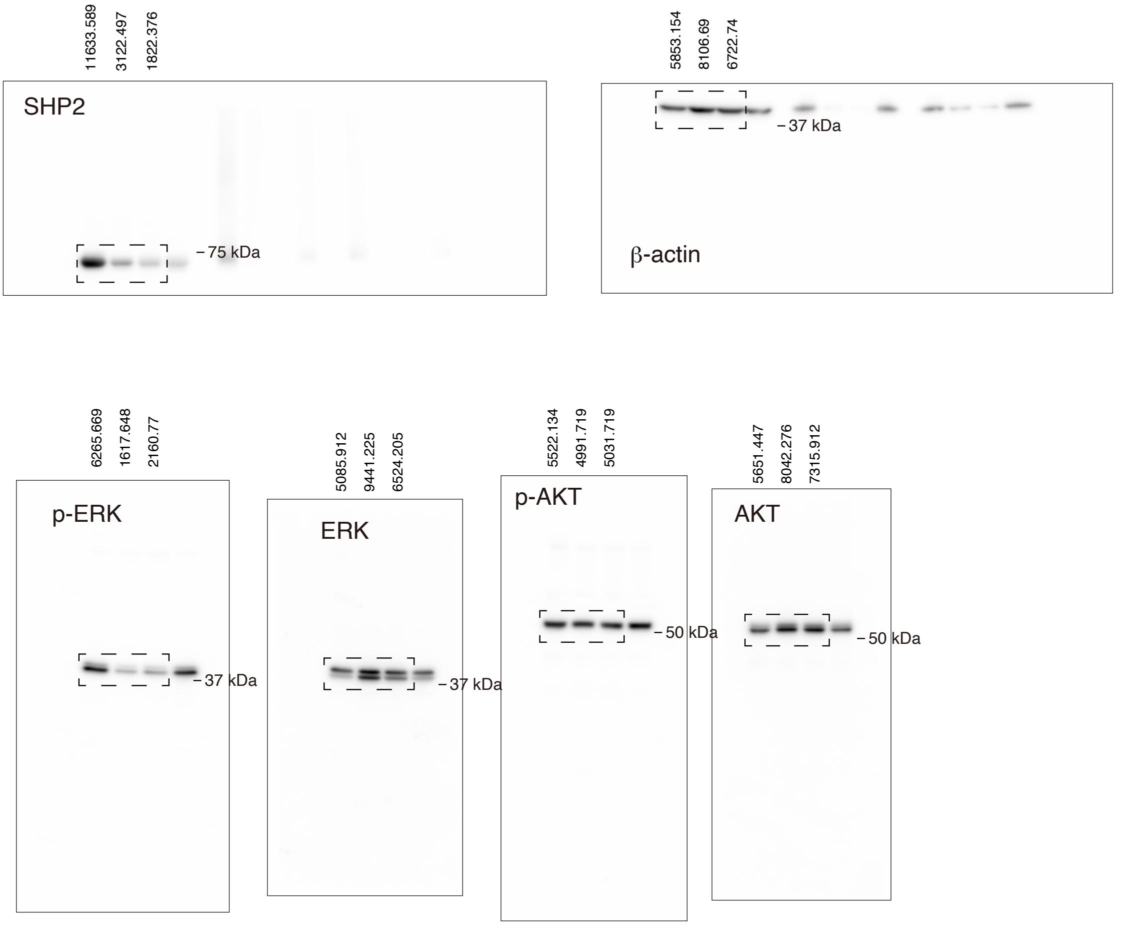

Full western blots for Figure 4C

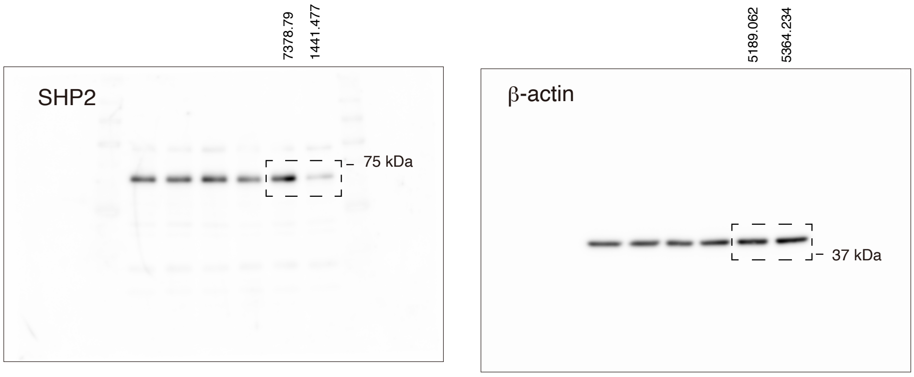

Full western blots for Figure 5B

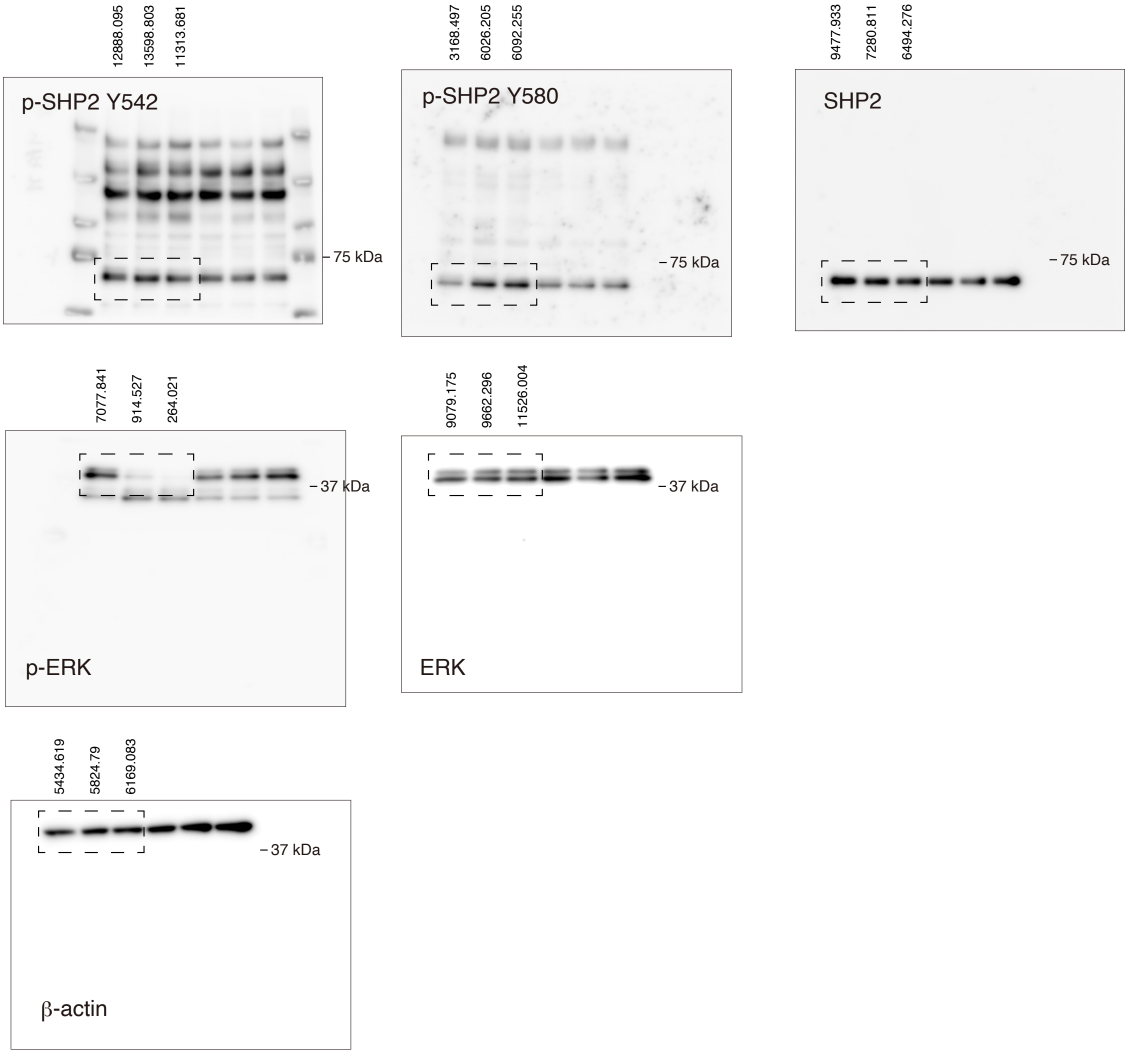

Full western blots for Figure 6B

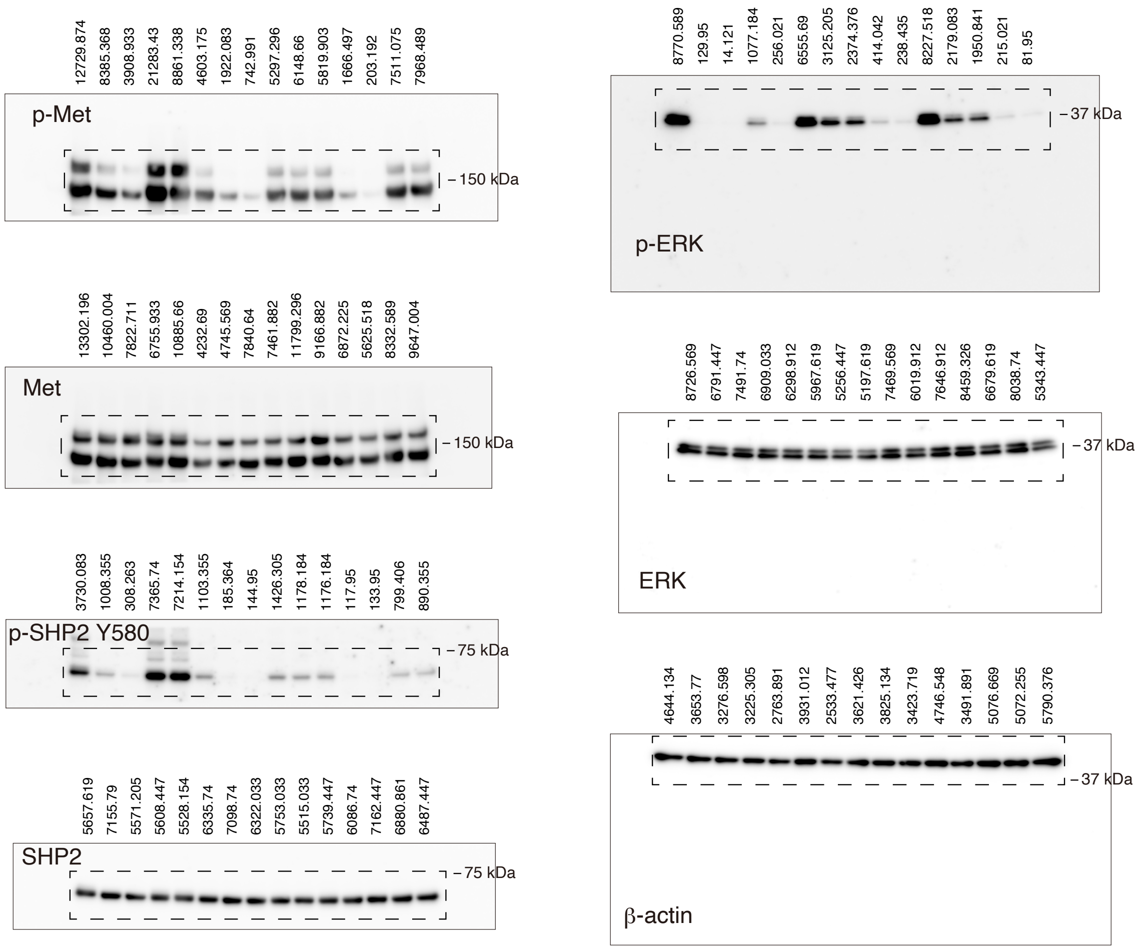

Full western blots for Figure 6E

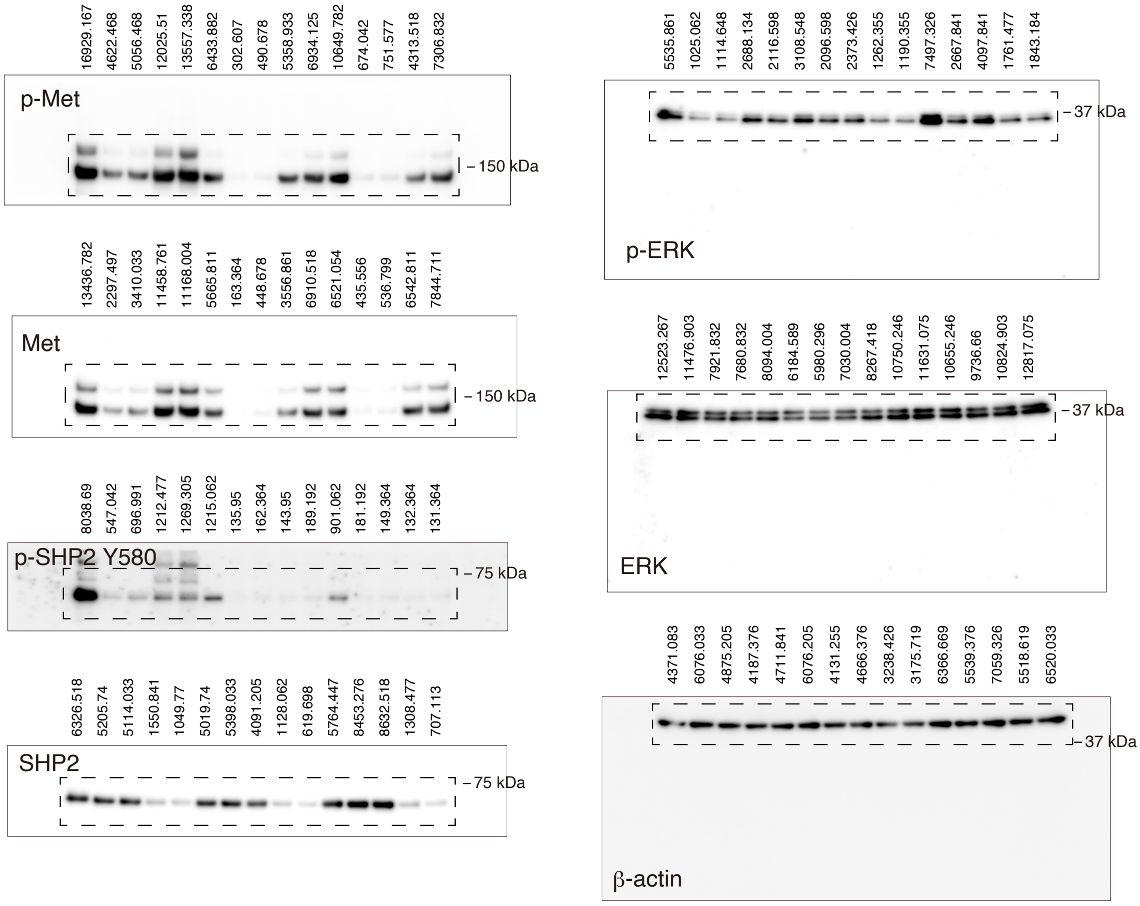

Full western blots for Figure S1A

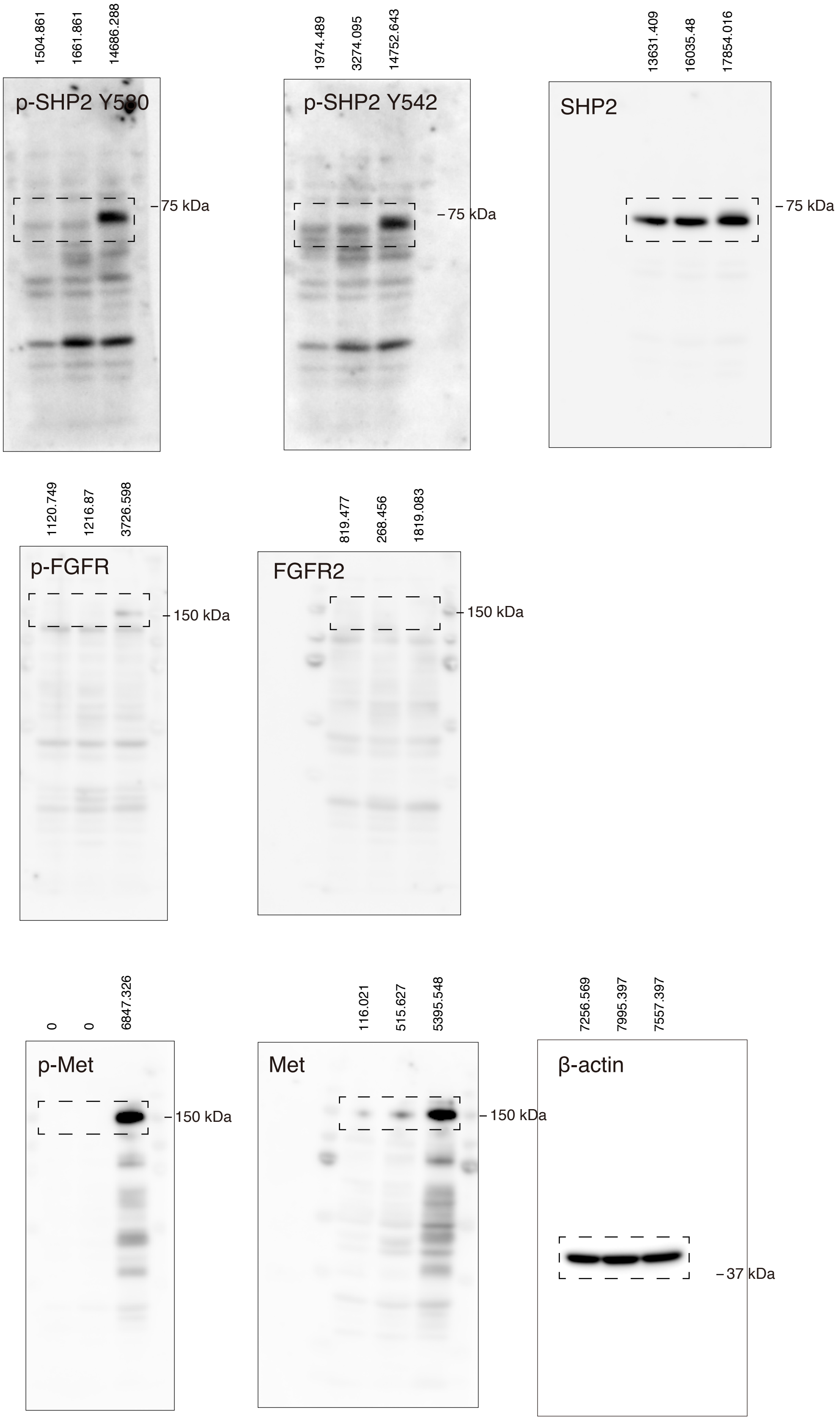

Full western blots for Figure S2

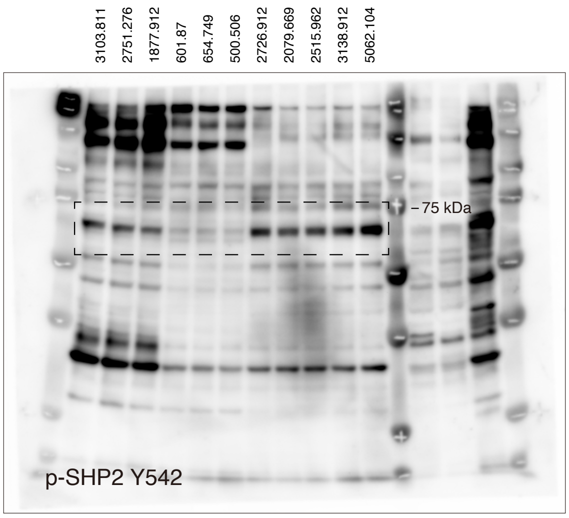

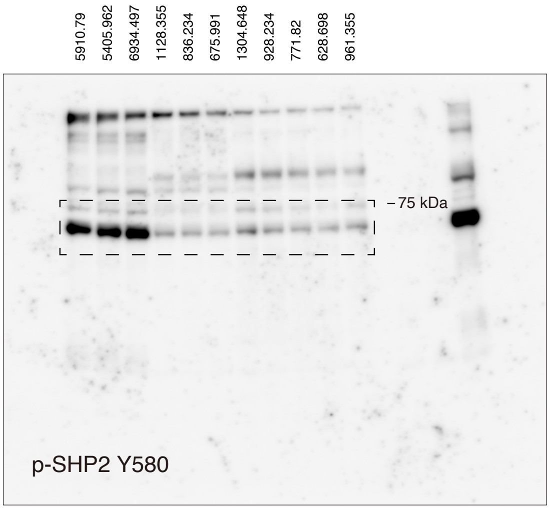

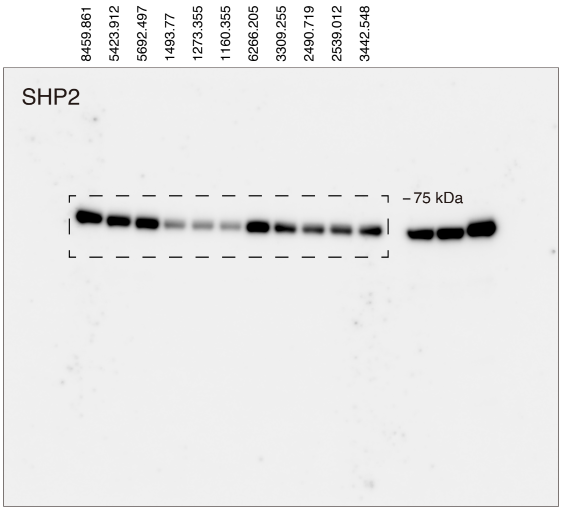

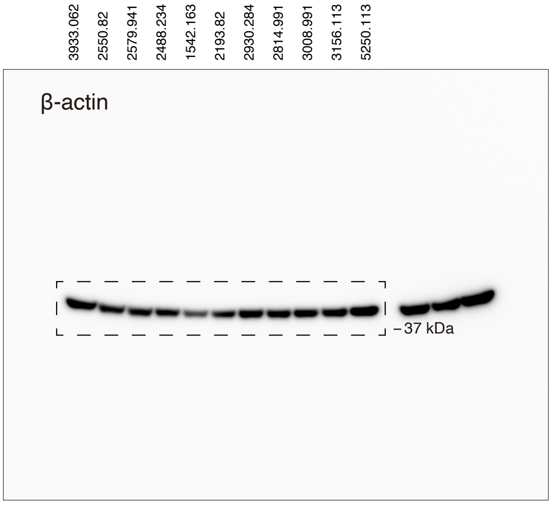

Full western blots for Figure S3

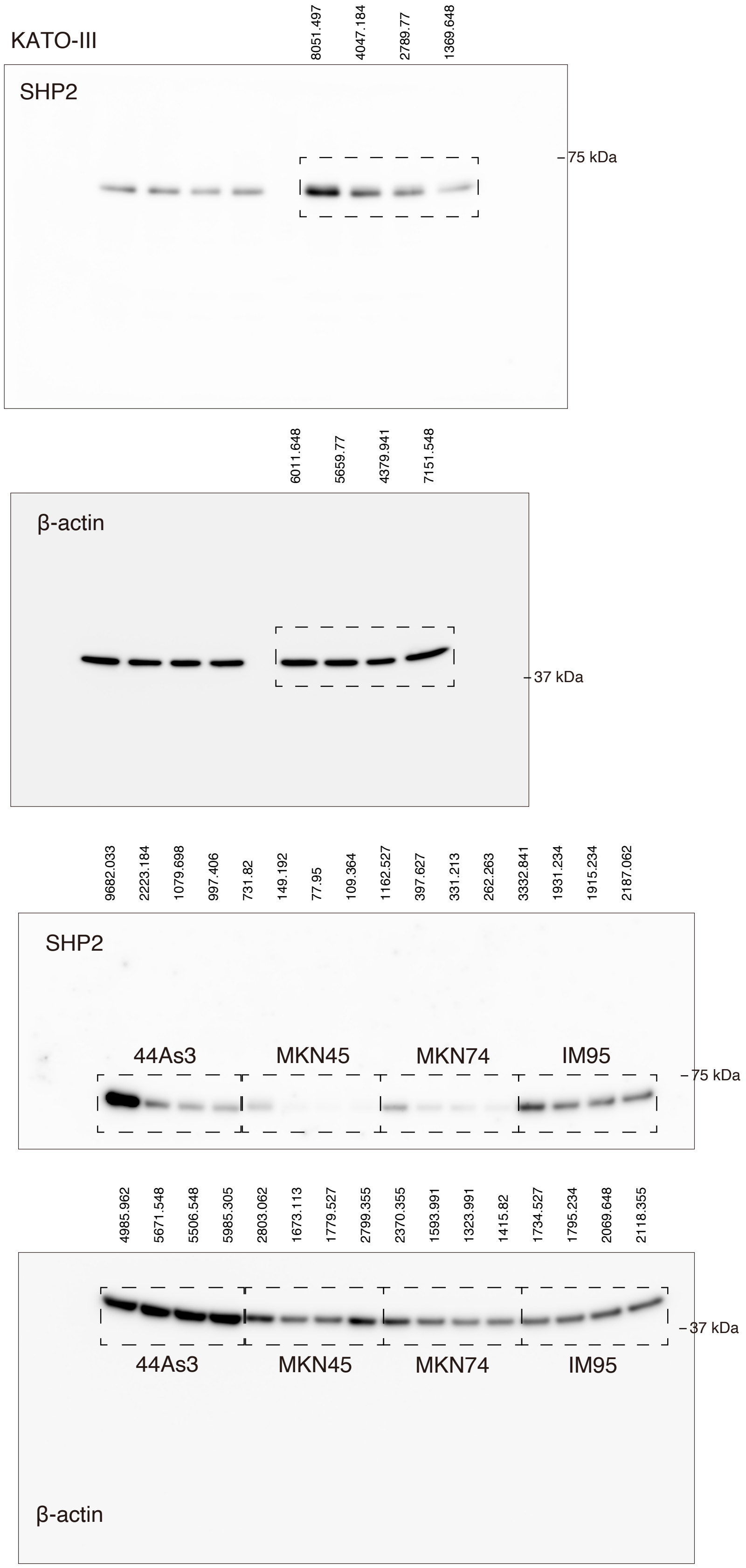

Supplement: Supplementary file 1 [file cancers-13-04309-s001.zip › File S1.pdf]
