# Supplementary material for: SHP2 as a Potential Therapeutic Target in Diffuse-Type Gastric Carcinoma Addicted to Receptor Tyrosine Kinase Signaling
Source: Cancers (Basel). 2021 Aug 26;13(17):4309. doi: 10.3390/cancers13174309 (PMC8430696; doi:10.3390/cancers13174309)
Supplement: Supplementary file 1 [file cancers-13-04309-s001.zip › Supplementary Figures.pdf]

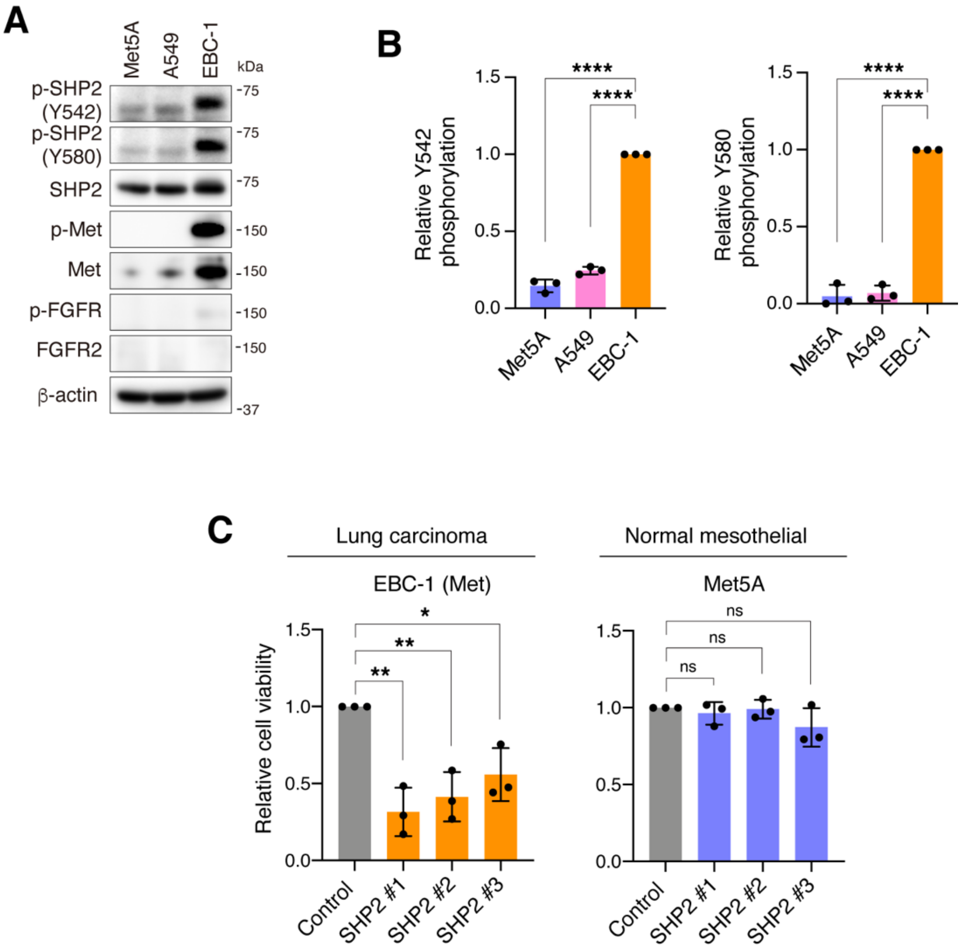

Figure S1. SHP2 phosphorylation and knockdown in lung cancer and mesothelial cell lines. (**A**) SHP2 phosphorylation in Met5A normal mesothelial cell line, A549 lung cancer cell line, and EBC-1 lung cancer cell line with Met gene amplification was examined by immunoblot analysis. (**B**) Quantification of SHP2 phosphorylation. Bars, SD ($n = 3$). ****, $p < 0.0001$. (**C**) EBC-1 and Met5A cells were transfected with control or SHP2 siRNAs for 6 days and examined for their viability. Bars, SD ($n = 3$). *, $p < 0.05$; **, $p < 0.01$, ns = not significant.

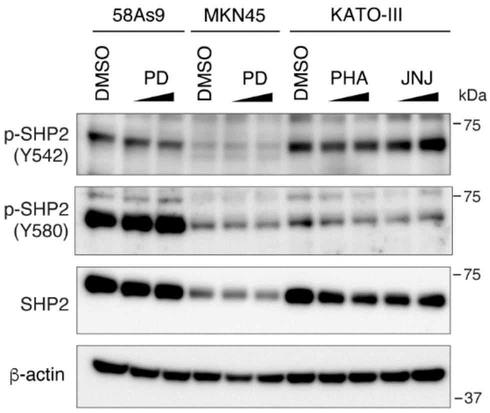

Figure S2. Immunoblot analysis of SHP2 phosphorylation in gastric carcinoma cells treated with inhibitors. 58As9 and MKN45 cells treated with FGFR inhibitor PD-173074 (PD), and in KATO-III cells treated with Met inhibitors PHA-665752 (PHA) and JNJ-38877605 (JNJ), at 100 or 300 nM for 2 h, were subjected to immunoblot analysis.

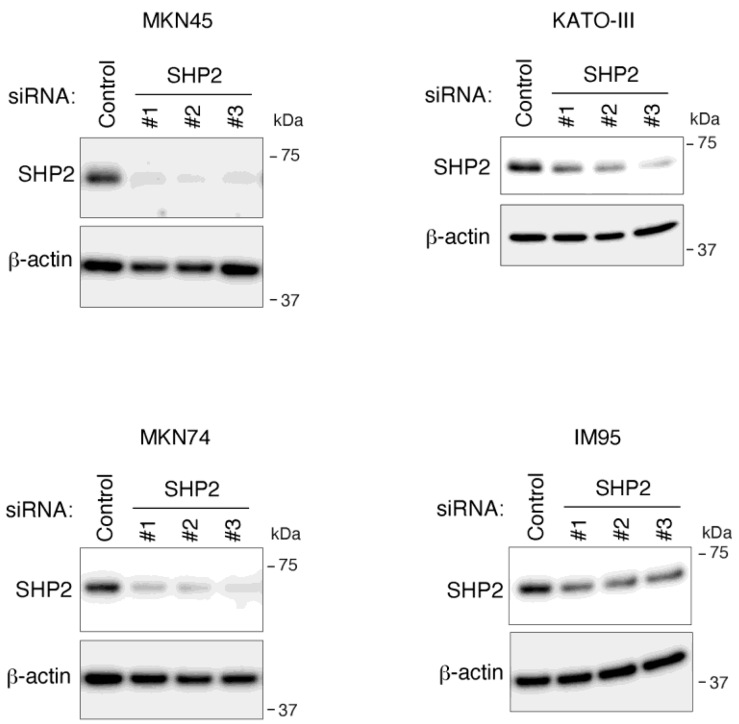

Figure S3. Immunoblot analysis of SHP2 expression in gastric carcinoma cells transfected with control or SHP2 siRNAs.

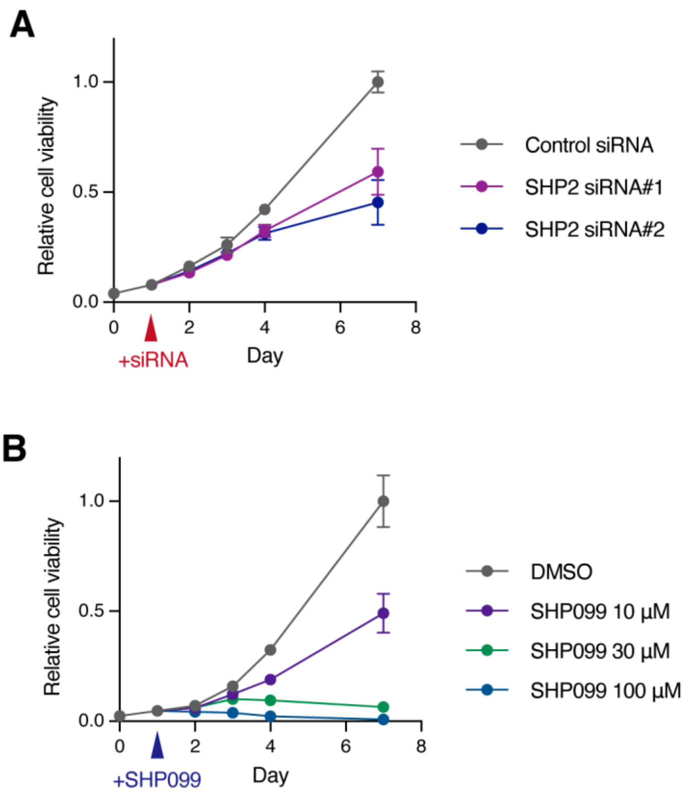

Figure S4. Time-course of cell viability after SHP2 siRNA transfection or inhibitor treatment. (**A**) 58As9 cells were transfected with control or SHP2 siRNAs and examined for their viability at the indicated time points. Bars, SD (n = 3). (**B**) 58As9 cells were treated with DMSO or SHP099 and examined for their viability at the indicated time points. Bars, SD (n = 3). Arrowheads denote the time points when siRNA or SHP099 was added.
